# Supplementary material for: Stochastic logistic models reproduce experimental time series of microbial communities
Source: eLife. 2020 Jul 20;9:e55650. doi: 10.7554/eLife.55650 (PMC7410486; doi:10.7554/eLife.55650)
Supplement: Supplementary file 1. — Analysis of all experimental data. Rank abundance, distribution of the differences between abundance at successive time points, neutrality tests and noise color. Supporting results. Code All python codes to perform time series simulations, analysis and make all different figures of the main paper and supplement are available at https://github.com/lanadescheemaeker/logistic_models (Descheemaeker and de Buyl, 2020; copy archived at https://github.com/elifesciences-publications/logistic_models). [file elife-55650-supp1.pdf]

Supplemental information to "Stochastic logistic models reproduce experimental time series of microbial communities"

Lana Descheemaeker[1,2] **and Sophie de Buyl**[1,2*]

***For correspondence:** Sophie.de.Buyl@vub.be (SdB)

[1]Applied Physics Research Group, Physics Department, Vrije Universiteit Brussel, Belgium; [2]Interuniversity Institute of Bioinformatics in Brussels, Vrije Universiteit Brussel - Université Libre de Bruxelles, Brussels, Belgium

Contents

Analysis of experimental data **2**
Rank abundance 2
Distribution of the differences between abundances at successive time points 2
Neutrality test 4
Noise color 4

Supporting results **4**
Validity of the neutrality measures 4
Examples of width distribution of ratios of abundances at successive time points 5
Discretizations of stochastic models with linear noise 6
Langevin discretization 6
Ricker discretization 6
Arato discretization 7
Discretizations of stochastic models with non-linear noise 9
Noise color and autocorrelation 9
The noise color depends on the product of the mean abundance and the self-interactions. 9
All noise characteristics can be obtained with the logistic model. 10
The noise characteristics remain for normalized time series. 12
The noise characteristics can also be obtained with generalized Lotka-Volterra equations. 12
The slope of the differences between abundances at successive time points $x(t+\delta t)/x(t)$ can be fine-tuned by a balanced combination of extrinsic and intrinsic noise. 12
The width of the distribution of ratios of abundances at successive time points increases with increasing strength of the noise. 14
Neutrality of generalized Lotka-Volterra models 14
Rounding time series to integer numbers makes the noise color lighter. 14
The self-organized instability model can be reproduced by the stochastic generalized Lotka-Volterra model. 17

Analysis of experimental data

We studied time series of different microbial communities. References for all of the time series can be found in ***Table 1***.

Table 1. References for all time series and compositions of microbial communities.

Label	Label in Figure 1 of the main article	Source
Stool A	Microbiome stool	Subject A gut of ***David et al.*** (***2014***)
Stool B		Subject B gut of ***David et al.*** (***2014***)
Plankton bacteria		Bacteria relative abundance of ***Martin-Platero et al.*** (***2018***)
Plankton eukarya	Plankton eukarya	Eukaryota relative abundance of ***Martin-Platero et al.*** (***2018***)
Female feces		Feces microbiome of subject F4 at the genus level (L6) ***Caporaso et al.*** (***2011***)
Male feces		Feces microbiome of subject M3 at the genus level (L6) ***Caporaso et al.*** (***2011***)
Female left palm	Microbiome palm	Left palm microbiome of subject F4 at the genus level (L6) ***Caporaso et al.*** (***2011***)
Male left palm		Left palm microbiome of subject M3 at the genus level (L6) ***Caporaso et al.*** (***2011***)
Female right palm		Right palm microbiome of subject F4 at the genus level (L6) ***Caporaso et al.*** (***2011***)
Male right palm		Right palm microbiome of subject M3 at the genus level (L6) ***Caporaso et al.*** (***2011***)
Female tongue	Microbiome tongue	Tongue microbiome of subject F4 at the genus level ((L6) ***Caporaso et al.*** (***2011***)
Male tongue		Tongue microbiome of subject M3 at the genus level (L6) ***Caporaso et al.*** (***2011***)
Sanger		Feces composition of DA-AD-1 of ***MetaHIT Consortium (additional members) et al.*** (***2011***)
Illumina		Feces composition of MH0001 of ***MetaHIT Consortium (additional members) et al.*** (***2011***) (original data ***MetaHIT Consortium et al.*** (***2010***))
Pyroseq		Feces composition of TS1_V2_turnbaugh of ***MetaHIT Consortium (additional members) et al.*** (***2011***) (original data ***Turnbaugh et al.*** (***2009***))

Rank abundance

Rank abundances of all studied time series can be found in ***Figure 1***. In nature, the abundance distributions are often power law, lognormal, or logarithmic series distributions. The abundance distribution of the microbial communities we study fits best a lognormal distribution (***Figure 2***).

Distribution of the differences between abundances at successive time points

We study the differences between abundances at successive time points of the time series in two ways. First, we look at the mean absolute value of the difference between abundances at successive time points $\langle| x(t+\delta t) - x(t) |\rangle$ and second we fit the distribution of the ratios of abundances at successive time points $x(t+\delta t)/x(t)$ with a lognormal. More details about the latter can be found in Examples of width distribution of ratios of abundances at successive time points . The slope of the mean absolute difference $\langle| x(t+\delta t) - x(t) |\rangle$ as a function of the mean abundance in the log-log scale ranges between 0.84 and 0.99 (***Figure 3***). The order of magnitude of the width of the distribution of the ratios of abundances at successive time points $x(t+\delta t)/x(t)$ is one and there is no correlation between the mean abundance and the width of this distribution (***Figure 4***). The goodness of the fit is characterized by the p-value of the Kolmogorov-Smirnov test. A higher p-value corresponds to a better fit of the distribution to a lognormal function (see Examples of width distribution of ratios of abundances at successive time points). The p-value is represented by the color of the points.

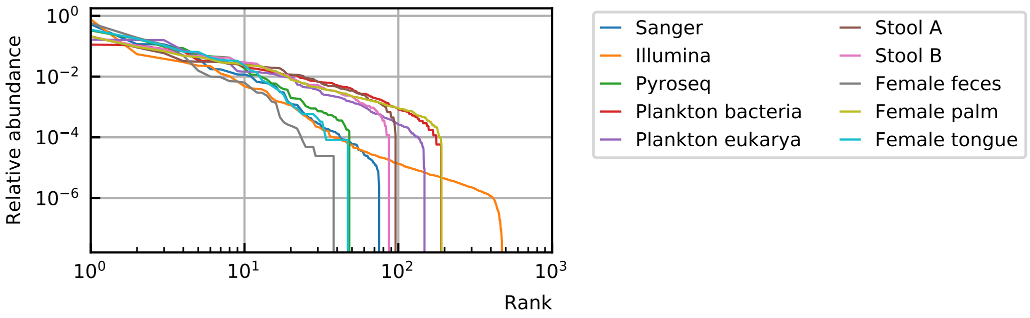

Figure 1. The rank abundance curve of the heavy-tailed abundance distribution of experimental measurements of microbial communities, from human microbiomes to plankton.

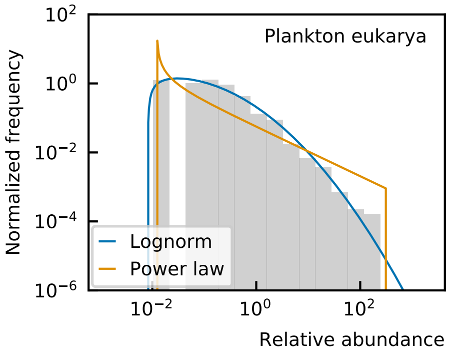

Figure 2. The abundance distribution of experimental data is heavy-tailed. It fits the lognormal distribution better than the power law.

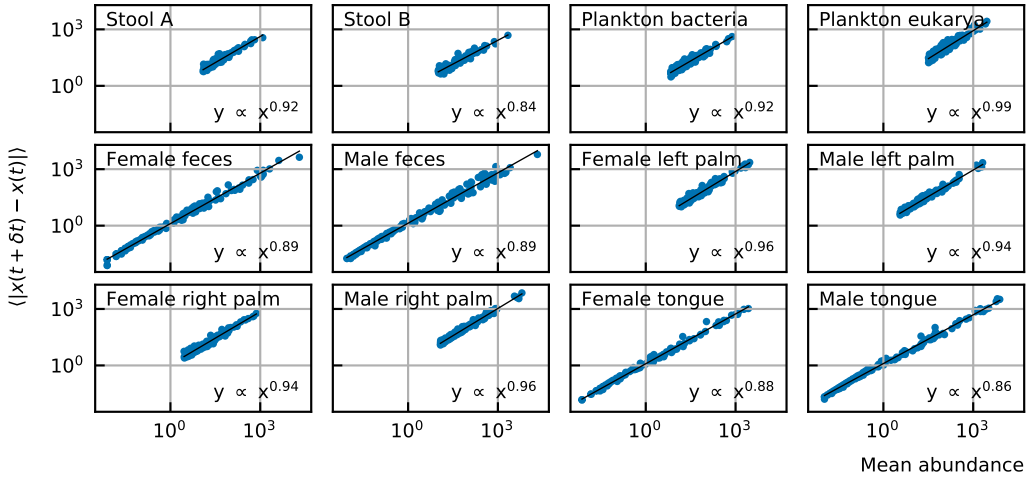

Figure 3. For experimental time series, the slope of the mean absolute difference between abundances at successive time points as a function of the mean abundance in the log-log scale ranges between 0.84 and 0.99.

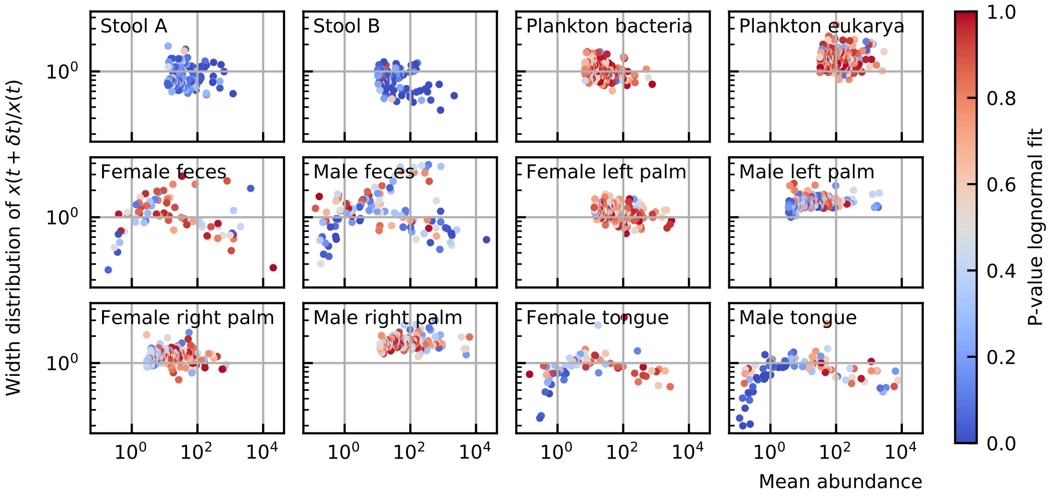

Figure 4. For experimental time series, the width of the distribution of the ratios of abundances at successive time points $x(t + \delta t)/x(t)$ is in the order of 1, which means that the fluctuations are large.

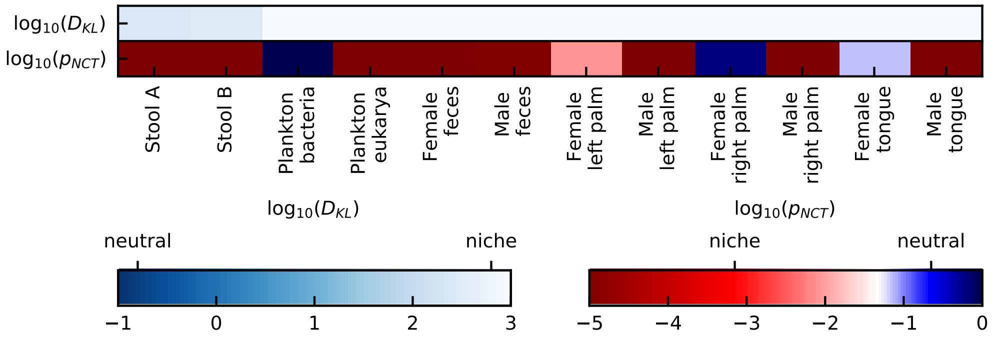

Figure 5. The values of the Kullback-Leibler divergence (KL) and the p-value of the neutral covariance test (NCT) are represented for the different microbial communities. The value can be read from the colorbars at the bottom. The neutral regime is given by a dark blue color. The niche regime is represented by a light colors for the KL and red for the NCT. For the NCT scale, a p-value of 0.05 is represented by white. Both neutrality tests show that most of the experimental time series are in the niche regime.

Neutrality test

Both neutrality tests, the Kullback-Leibler divergence and the p-value of the neutral covariance test, show that most of the experimental time series are in the niche regime (***Figure 5***).

Noise color

The noise color is predominantly in the pink to white region. It is also independent of the mean abundance (***Figure 6***).

Supporting results

Validity of the neutrality measures

One way of generating a neutral time series is by considering a lattice of N individuals, in which, every time step, one individual is replaced by another. Each individual of the lattice has an equal probability of being replaced (the probability is N^{-1}). The disappearance of the first species can be interpreted as the result of either death or emigration. The replacing individual is either the result of immigration or growth. The probability of immigration depends on the immigration rate λ $(0 \leq \lambda \leq 1)$. In case of an immigration event, all species of the external species pool S have an equal probability of immigrating. The probability of a growth event is given by the remaining $1 - \lambda$. In

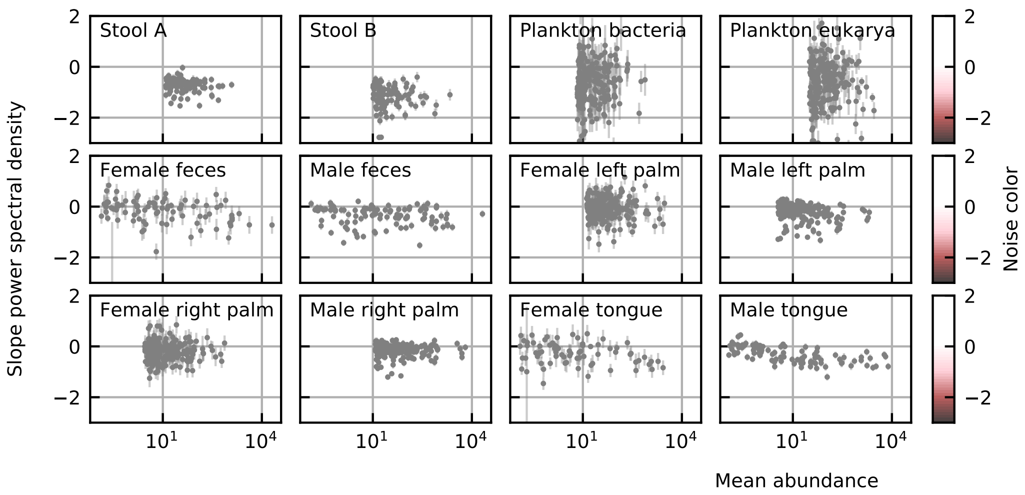

Figure 6. For experimental time series, the noise color is predominantly in the pink to white region. It is also independent of the mean abundance.

case of growth, every individual has an equal probability of growing. Time series generated in this way depend on three variables: the length of the simulation time T, the immigration probability λ, and the number of individuals N. We study the effect of these three variables on both neutrality measures (***Figure 7***).

As discussed in ***Wennekes et al.*** (***2012***), the neutral-niche debate is a scale problem. Looking at a process from close-by and describing all the details, results in the niche regime. Dynamics of large systems where the details are not modeled appear to be dominated by random processes. The questions are: at which scale is one studying the process and which of the deterministic or stochastic processes is dominating? We can see this in the upper row of ***Figure 7***A. Larger immigration gives rise to a neutral regime. However, for short time series and large communities, one will only see the "details" because the time series is not yet in steady-state. Consequently, this results in niche values. In the absence of immigration, the "winner-take-all" principle holds. During a transient period, all species but one go extinct in a stochastic manner. In the end, the steady-state consists of only one species surviving. The duration of the transient depends on the size of the community. This is why the time series are in the niche regime with respect to grouping in the absence of immigration when the time series is long enough (***Figure 7***B). For small immigration and small communities, the "winner" cannot take over due to the immigration of other species, but the most fit species dominate the community one by one in a random order. For short time series, there are only a few changes of dominating species and this apparent structure in the time series gives rise to a niche regime (***Figure 7***B). For longer time series however, there are many random changes of the dominating species, which results in a neutral character (***Figure 7***B).

Examples of width distribution of ratios of abundances at successive time points

To study the jumps that the species abundances make over time, we consider the distribution of the ratios of abundances at successive time points $x(t+\delta t)/x(t)$. This distribution can be fitted to a lognormal function:

$$f(x) = \frac{1}{\sqrt{2\pi}sx}\exp\left(-\frac{\ln^2(x)}{2s^2}\right). \tag{1}$$

We impose the constraint that the median of this distribution is one because we assume that the time series fluctuates around steady-state and that there will be as many steps for which the abundance is increasing as there are steps for which the abundance is decreasing. We define the shape parameter s as the width of the step distribution. Some examples of fits are shown in ***Figure 8***. The goodness of the fit is obtained through the Kolmogorov-Smirnov test, of which we

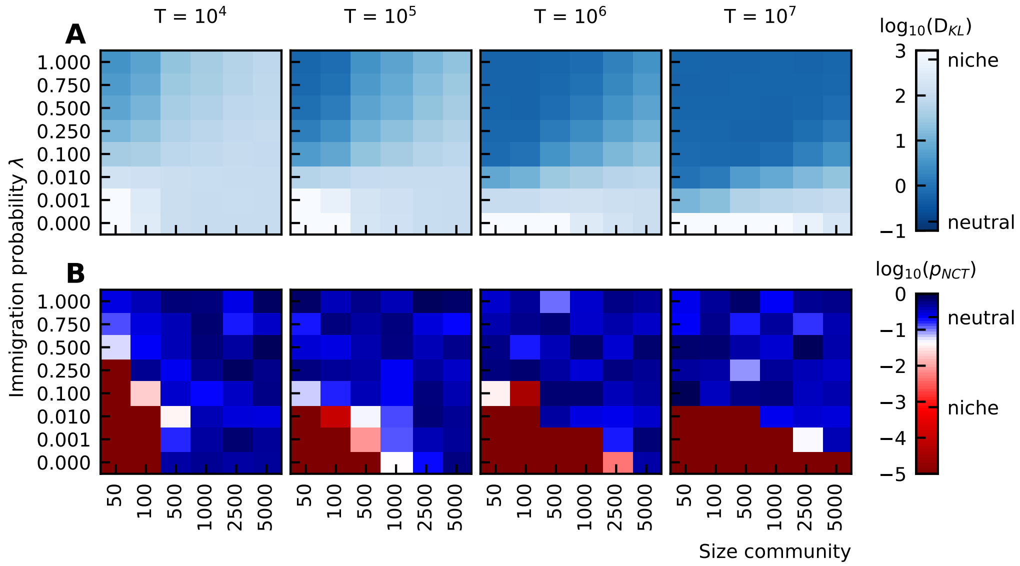

Figure 7. Neutrality tests, (A) Kullback-Leibler divergence and (B) the p-value of the neutral covariance test. We expect to see the neutral regime for all time series because they are generated by random birth, death and immigration events. For short time series and a large size of the community, only the details are seen and the result is niche. For small immigration, there is a "winner-take-all" effect, for which the neutrality covariance test, which tests for invariance with respect to grouping, gives a niche result.

represent the p-value. A higher p-value corresponds to a better fit of the distribution to a lognormal function. The p-value is represented by the color of the fitted line.

Discretizations of stochastic models with linear noise

The implementation of the linear multiplicative noise is as follows,

$$dx_i = \lambda_i dt + g_i x_i dt + \sum_j \omega_{ij} x_i x_j dt + x_i \sigma_i dW, \quad (2)$$

with dW an infinitesimal element of a Brownian motion which is defined by a variance of dt ($dW \sim \sqrt{dt}\mathcal{N}(0,1)$).

Because of its nonlinear nature, generalized Lotka-Volterra equations cannot be solved analytically. It is therefore necessary to perform simulations of the stochastic differential equations.

When we consider a linear multiplicative noise, we must first choose a discretization of the deterministic differential equations. Two possibilities can be found in the literature. The first one is the Ricker implementation and the second one we here call the Langevin implementation.

Langevin discretization

To solve the stochastic equations numerically, we can use the Euler-Maruyama method. Here the discretization becomes

$$x_i(t+\delta t) = x_i(t) + g_i x_i(t)\delta t + \omega_{ij} x_i(t) x_j(t)\delta t + \sigma \delta W x_i(t). \quad (3)$$

Due to the choice of discretization x can become zero or negative. Because this is the most straightforward discretization of the Langevin equation, we call it the Langevin discretization.

Ricker discretization

For this implementation, we assume there is no migration ($\lambda_i = 0$). First, we rewrite ***Equation 2*** as the derivative of the species abundance divided by the species abundance itself, such that we can replace the latter by a derivative of the logarithm of the species abundance:

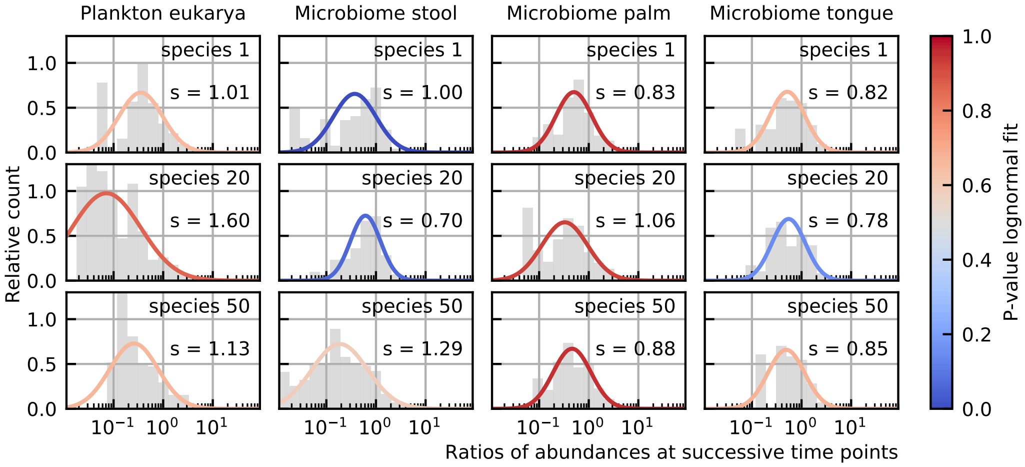

Figure 8. A lognormal function is fitted to the ratios of abundances at successive time points $x(t+\delta t)/x(t)$ for different species. The species number denotes its rank. We interpret the shape parameter s as a measure for the width of the steps. Here we see that this width s is of the order of 1 for all species. The goodness of the fit (p-value of the Kolmogorov-Smirnov test) is represented by the color of the fitted line. For most species the fit is good (high p-values).

$$\frac{\dot{x}_i}{x_i} = g_i + \sum_j \omega_{ij} x_j + \sigma \frac{dW}{dt}, \tag{4}$$

$$\dot{\ln x_i} = g_i + \sum_j \omega_{ij} x_j + \sigma \frac{dW}{dt}. \tag{5}$$

When dividing by the species abundance, we assumed that x_i can never become zero (or negative for the solution is continuous). This would be true in the theoretically continuous framework and in the absence of noise, but this is not what is observed in experimental time series where the abundances are discrete numbers and can become zero in the event of extinction.

Using the assumption $\delta t \ll 1$, we obtain

$$\ln x_{t+\delta t} - \ln x_t = \delta t(\omega x + g) + \sigma \delta W, \tag{6}$$

$$x_{t+\delta t} = x_t \exp \delta t(\omega x + g) \exp \sigma \delta W \tag{7}$$

The δW are normally distributed random elements ($\delta W \sim \sqrt{\delta t}\mathcal{N}(0,1)$).

Due to the choice of discretization, x_i can never become zero or negative, which is fundamentally different from the previous implementation.

Arato discretization

Using Ito calculus (see ***Box 1*** for a short introduction) a third discretization can be obtained:

$$x_i(t) = x_i(0) \exp \left(g_i t - \frac{\sigma_i^2 t}{2} - \sum_{j=1}^{n} \omega_{ij} \int_0^t x_j(s) ds + \sigma W_i(t) \right) \tag{8}$$

as calculated in ***Arató*** (***2003***).

In the limit of small noise or small timesteps, the Langevin, Ricker, and Arato discretizations are equal (***Figure 9***A). For large noise, a clear difference can be seen for both implementations (***Figure 9***B).

The parameters of the implementation, such as the discretization, length of the simulation, and integration time step dt are not influencing the results of the noise color (***Figure 9***C-E).

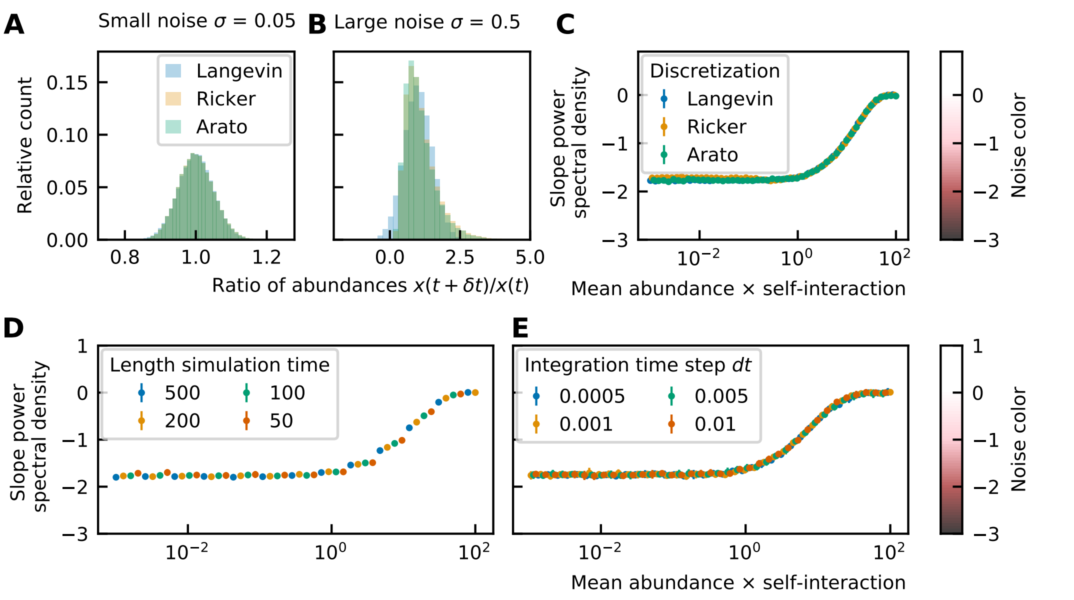

Figure 9. Distributions of x_{t+dt} for $x_t = 1$ for three different discretizations: Langevin $x_{t+dt} = x_t + x_t\sigma dW$, Ricker $x_{t+dt} = x_t \exp(\sigma dW)$ and Arato $x_{t+dt} = x_t \exp\left(\frac{-\sigma^2}{2}dt + \sigma dW\right)$ with $dW \sim \mathcal{N}(0,1)$ and $dt = \sigma^2$ for small noise $\sigma = 0.05$ (A) and large noise $\sigma = 0.5$ (B). For both these figures the sampling time step δt is equal to the integration time step dt. For small noise, all implementations result in the same abundance distribution. For large noise, the distributions are different. The Langevin implementation allows abundances to become zero and negative (which will be equalled to zero). The abundances of the Ricker and Arato implementation never become zero. The noise color does not depend on the particular implementation: it is independent of the discretization (C), length of the time series (D) and integration time step dt (E).

Box 1. Basics of Ito calculus

A Brownian motion or Wiener process is described by a probability distribution over the set of continuous functions $B : \mathbb{R}_{\geq 0} \to \mathbb{R}$ which is defined by three characteristics:

1. $P(B(0) = 0) = 1$, the motion starts at the origin,
2. the motion is stationary: $\forall 0 \leq s \leq t\,:\, B(t) - B(s) \sim \mathcal{N}(0, t-s)$,
3. the increments are independent: if intervals $[s_i, t_i]$ are not overlapping than $B(t_i) - B(s_i)$ are independent.

An important feature of such a motion is the quadratic variation, which states that the expectation value of dW^2 is dt. In a regular derivative, the square and other higher-order terms of the infinitesimal dt of the Taylor expansion are ignored. Due to the quadratic variation, the square term of dW cannot be ignored and Ito's lemma states that for a stochastic process X_t:

$$dX_t = \mu dt + \sigma dW_t, \tag{9}$$

and for f a smooth function, we have that

$$df(t, X_t) = \left(\frac{\partial f}{\partial t} + \mu\frac{\partial f}{\partial x} + \frac{1}{2}\sigma^2\frac{\partial^2 f}{\partial x^2}\right)dt + \frac{\partial f}{\partial x}dW_t, \tag{10}$$

and as a consequence,

$$d(\ln x_i) = \frac{dx_i}{x_i} - \frac{(dx_i)^2}{2x_i^2}. \tag{11}$$

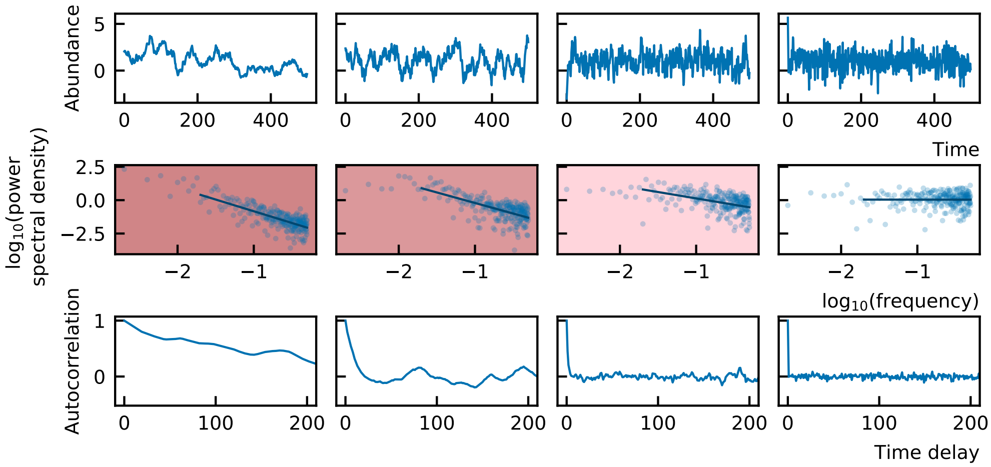

Figure 10. Different stochastic time series (first row) with the corresponding power spectral density (second row) and autocorrelation functions (last row). The background color of the power spectral density corresponds to the color of the noise. When there is more structure present in the time series, the autocorrelation function is less steep at small time delays and the noise is darker.

Discretizations of stochastic models with non-linear noise

In the main manuscript, we discussed choosing a noise term that is linear in the species abundance because this represents best the external noise on growth and death of the species. Intrinsic noise (due to discreteness) could be represented by a term that is proportional to the square root of the species abundance (sqrt multiplicative noise) (***Walczak et al., 2012***). In this case, the discretization is:

$$x_i(t+\delta t) = x_i(t) + g_i x_i(t)\delta t + \omega_{ij} x_i(t) x_j(t)\delta t + \sigma \delta W \sqrt{x_i(t)}. \tag{12}$$

We also considered a constant noise term (additive noise), which represents noise due to stochastic immigration and emigration of species. The corresponding discretization is:

$$x_i(t+\delta t) = x_i(t) + g_i x_i(t)\delta t + \omega_{ij} x_i(t) x_j(t)\delta t + \sigma \delta W. \tag{13}$$

In the main paper, it is shown that the source of the noise, linear multiplicative, square root multiplicative, or additive, has no influence on the relation between the mean abundance, self-interaction, and noise color.

Noise color and autocorrelation

For any time series, an autocorrelation function can be calculated. The latter is obtained by calculating the correlation of a time series with the same time series starting at a later time point. The difference in time between the starting point of both time series is the delay. The autocorrelation function is defined as the autocorrelation for all positive delay values. As explained in the supplemental material of ***Faust et al.*** (***2018***), the noise color is related to the autocorrelation of the time series. The lighter the noise color is, the steeper the autocorrelation function is for small time delays. For dark noise, the autocorrelation decreases more slowly and the time series is said to contain more structure. An illustration of this is given in ***Figure 10***.

The noise color depends on the product of the mean abundance and the self-interactions.

To study the noise color, we represented it as a function of different variables. We conclude that the noise color depends on the product of the mean abundance and the self-interaction (***Figure 11***). Given the sampling rate, this curve can be fitted by a sigmoid with the x-axis in the log scale

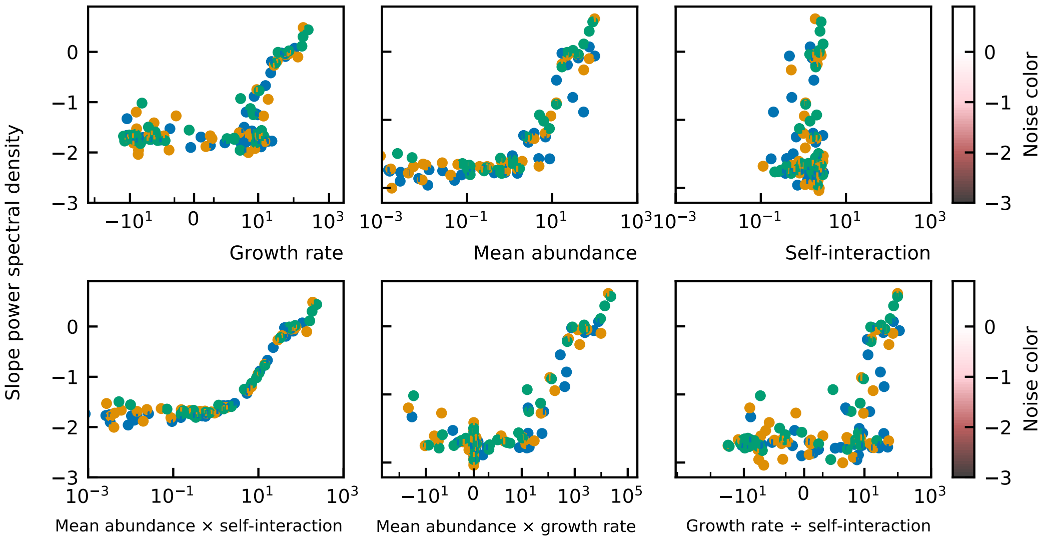

Figure 11. The noise color depends on the product of the mean abundance and the self-interaction. The correspondence with the growth rate or mean abundance are less pronounced.

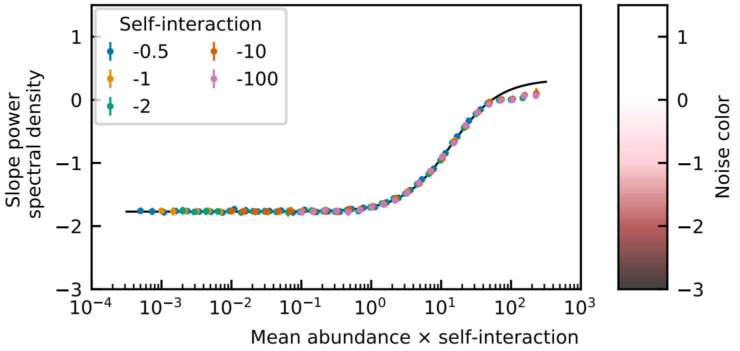

Figure 12. The curve of the noise color as a function of the logarithm of the mean abundance and self-interaction can be fitted with a sigmoid function.

(***Figure 12***) and the fitted function can be used to calculate the self-interaction of a species given its noise color and mean abundance.

All noise characteristics can be obtained with the logistic model.

Given the abundance and the slope of the power spectral density (noise color), we can determine the self-interaction and growth rate of a species via the fitted sigmoid curve (***Figure 13***). The fitted sigmoid curve gives the value of the product of the mean abundance and self-interaction given the slope of the power spectral density (***Figure 13***B). Using this value and the relation between the self-interaction and the product of the self-interaction and mean abundance (colored lines of ***Figure 13***F), the self-interaction can be calculated (***Figure 13***E). For noninteracting species, the growth rate equals the product of the mean abundance and self-interaction (black line in ***Figure 13***H). Therefore, the growth rate is retrieved immediately (***Figure 13***G).

Once all the parameters are determined, we can perform simulations with large linear multiplicative noise that have the same characteristics as experimental time series. These results are presented in Figure 4 of the main paper.

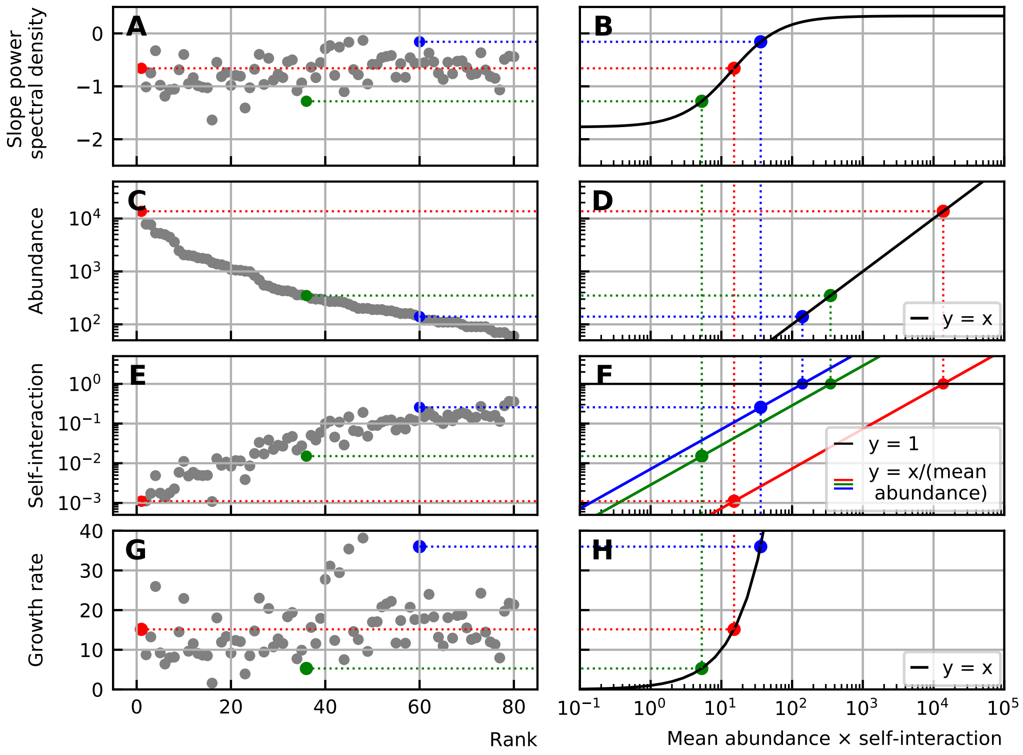

Figure 13. This scheme represents how the self-interaction and growth rate can be retrieved from the noise color and abundance data. For a given sampling step δt, the relation between the noise color, abundance and self-interaction can be fitted by a sigmoid curve (B). Given the abundance (D), the relation between the mean abundance and self-interaction can be drawn (colored lines in F). The value of the noise color can then be translated to a value of the self-interaction (A-B-F-E). For noninteracting species, the growth rate equals the product of the mean abundance and self-interaction. The growth rate is thus easily determined (A-B-H-G).

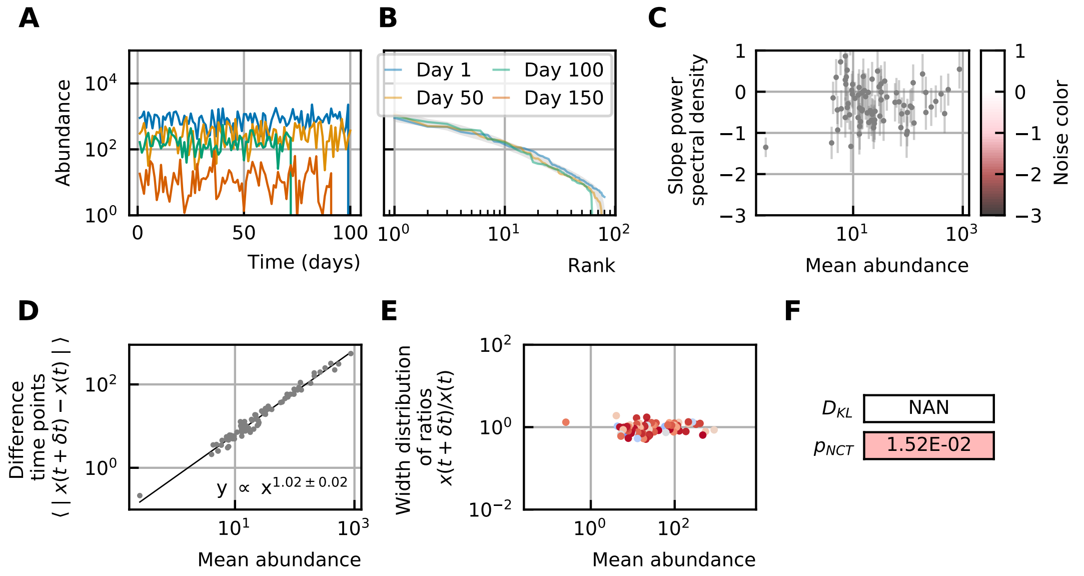

Figure 14. All properties of the noise remain after normalization: (A) Time series. (B) The rank abundance of a heavy-tailed abundance distribution remains stable over time. (C) Noise color in the white-pink region with no dependence on the mean abundance. (D) The slope of the mean absolute difference between abundances at successive time points is around 1. (E) The width of the distribution of the ratios of abundances at successive time points is in the order of 1 and independent of the mean abundance. (F) Results of the neutrality test in the niche regime.

The noise characteristics remain for normalized time series.

It is experimentally often challenging to determine the absolute abundances of species. Therefore, only fractional abundances are measured. The logistic equation models the absolute abundance of a species. In ***Figure 14***, we show that normalizing the species abundances for every time point does not influence the characteristics.

The noise characteristics can also be obtained with generalized Lotka-Volterra equations.

In the presence of inter-species interactions, the properties of the experimental time series can also be retrieved. In ***Figure 15***, an example is given with interaction strength 0.02 ($\omega_{ij} \propto \mathcal{N}(0, 0.02)$) and connectance 0.1 which means that 90% of the interactions are set to 0. The rank abundance and self-interactions are derived from the stool A data and the noise is linear with strength $\sigma_{\text{lin}} = 2.5$. The growth rate is calculated given the steady-state abundances and interaction matrix. Interactions are however not necessary to obtain the characteristics, as seen in the main text. The same results still hold when normalizing the time series (***Figure 16***).

The slope of the differences between abundances at successive time points $x(t+\delta t)/x(t)$ can be fine-tuned by a balanced combination of extrinsic and intrinsic noise.

We can combine extrinsic (linear multiplicative) and intrinsic (square root multiplicative) noise such that the slope of the absolute differences between abundances at successive time points as a function of the mean abundance in the log-log space is smaller than one as observed in the experimental time series. For linear multiplicative noise, the slope has a value around 1, for sqrt multiplicative noise the value is around 0.66 (***Figure 17***). A combination where the strengths of both noise sources are equal ($\sigma_{\text{lin}} = \sigma_{\text{lin}} = 0.5$) results in an intermediate slope (***Figure 17***).

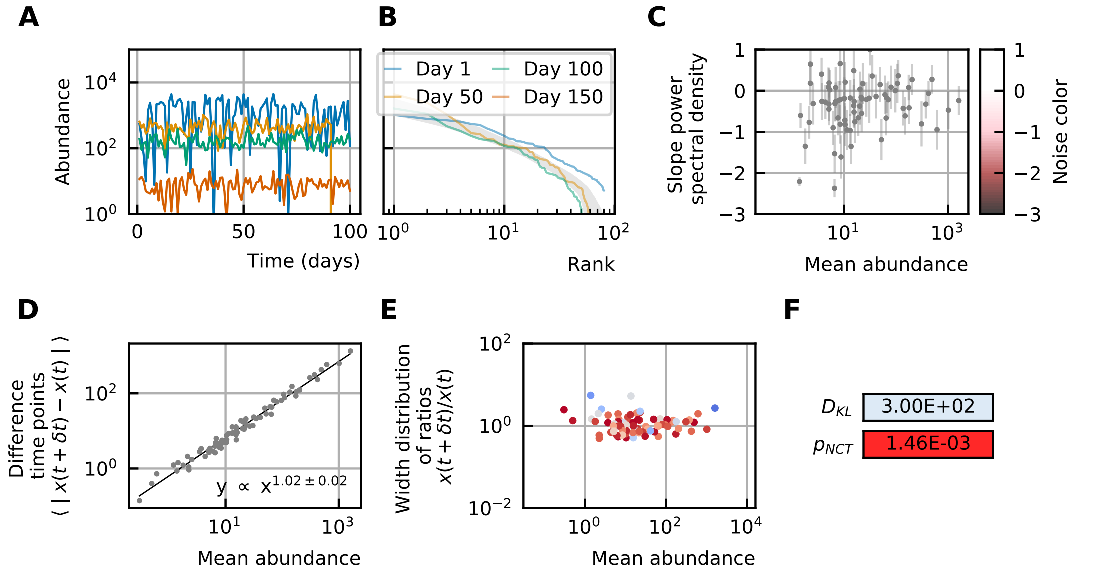

Figure 15. All properties of the noise can still be obtained in the presence of interactions: (A) Time series. (B) The rank abundance of a heavy-tailed abundance distribution remains stable over time. (C) Noise color in the white-pink region with no dependence on the mean abundance. (D) The slope of the mean absolute difference between abundances at successive time points is around 1. (E) The width of the distribution of the ratios of abundances at successive time points is in the order of 1 and independent of the mean abundance. (F) Results of the neutrality test in the niche regime.

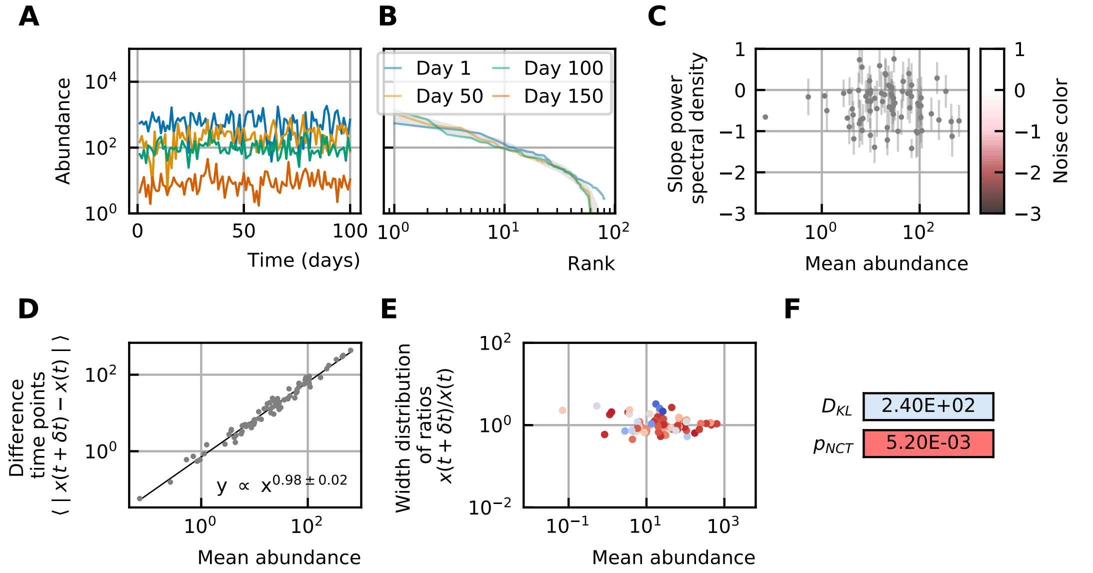

Figure 16. All properties of the noise can still be obtained in the presence of interactions and when the time series are normalized: (A) Time series. (B) The rank abundance of a heavy-tailed abundance distribution remains stable over time. (C) Noise color in the white-pink region with no dependence on the mean abundance. (D) The slope of the mean absolute difference between abundances at successive time points is around 1. (E) The width of the distribution of the ratios of abundances at successive time points is in the order of 1 and independent of the mean abundance. (F) Results of the neutrality test in the niche regime.

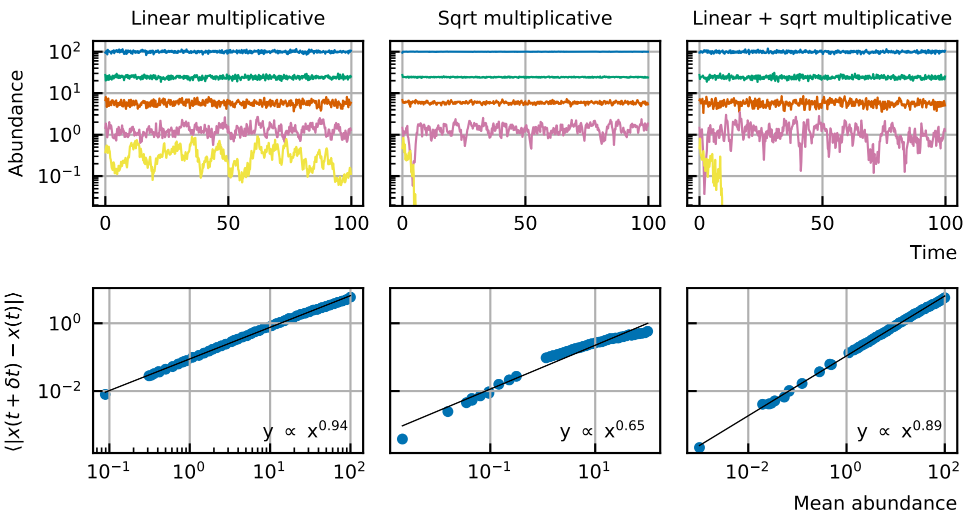

Figure 17. The slope of the mean absolute difference between abundances at successive time points with respect to the mean abundance in log-log scale is around 1 (left). For square root noise, this slope is around 0.66 (middle). A mix of linear and square root noise results in intermediate slopes (right). Here, a combination of linear and square root multiplicative noise with equal strengths ($\sigma_{\text{lin}} = \sigma_{\text{sqrt}} = 0.5$) was used to obtain a slope of 0.89.

The width of the distribution of ratios of abundances at successive time points increases with increasing strength of the noise.

The distribution of the ratios of abundances at successive time points $x(t+\delta t)/x(t)$ depends on the rank abundance, self-interaction, and interaction strengths. In a first implementation, we simulate 50 interacting species with linear noise. The interaction matrix has elements drawn from a normal distribution with mean 0 and the standard deviation given by the interaction strength. All self-interactions are imposed to be -1. For an increasing noise strength σ_{lin}, the width of the distribution of ratios of abundances at successive time points $x(t+\delta t)/x(t)$ increases (***Figure 18***). In a second implementation, we use 50 species with the rank abundance of the experimental human stool data (Stool A) and self-interactions inferred from the noise color as explained in the main paper. The interaction matrix for the interacting case is much more sparse here, the connectance is 0.1 which means that only 10% of the interactions are non-zero. The non-zero interactions are distributed like a normal distribution with mean 0 and a standard deviation of 0.03. As expected, increasing the strength of the noise increases the width of the distribution, and adding interaction also slightly increases the width (***Figure 19***).

Neutrality of generalized Lotka-Volterra models

The higher the interaction strength, the more niche the time series become (***Figure 20***).

Rounding time series to integer numbers makes the noise color lighter.

For low abundances, there is a finite precision effect. When we calculate the noise color for the time series with a precision up to the integer, we see that the noise color becomes lighter for smaller abundances (***Figure 21***). This is consistent with the fact that the noise color is correlated with the autocorrelation and the amount of structure in the time series, i.e. the predictability of the time series. In the time series that have smaller precision, there is less information and the time series is less predictable, therefore the noise color is lighter.

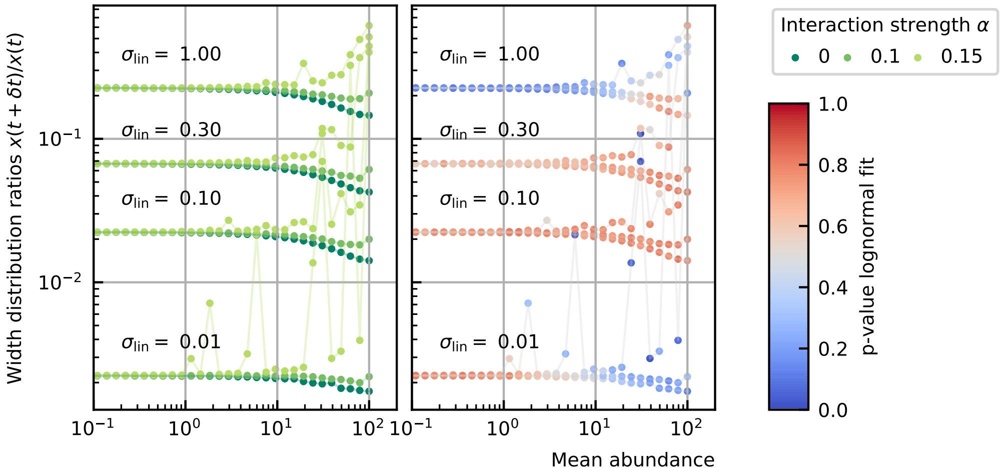

Figure 18. The width of the distribution of the ratios of abundances at successive time points $x(t + \delta t)/x(t)$ for time series of 50 interacting species with equal abundances increases with increasing strength of the linear noise. For high abundances, the width also increases with the interaction strength.

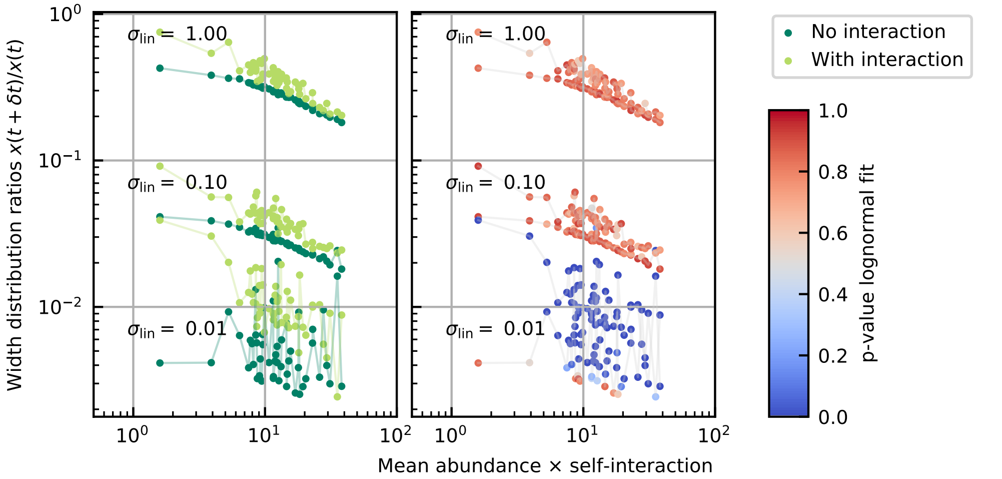

Figure 19. The width of the distribution of the ratios of successive time points for time series for 50 interacting species with a rank abundance and inferred self-interaction of the stool A data. The width increases with increasing strength of the linear noise.

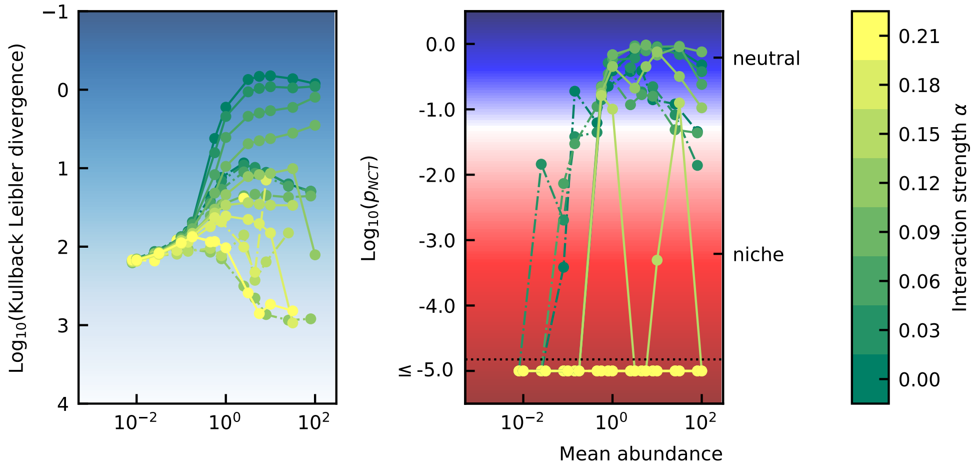

Figure 20. The higher the interaction strength the more niche the time series become.

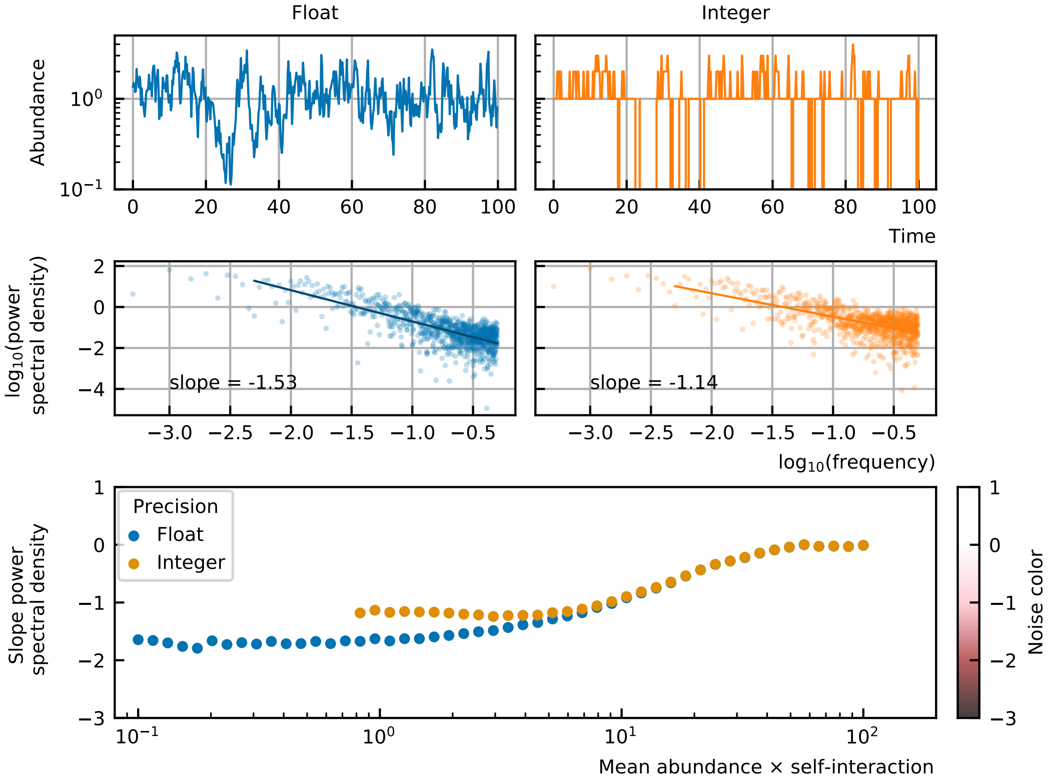

Figure 21. Finite precision makes the noise color lighter for species with small abundances.

Table 2. Parameter values of varied parameters.

Column in figure 1	1	2	3
Varying parameter	Immigration rate	Extinction rate	Interaction strength
Immigration (m)	$\mathcal{U}$(0, immigration)	$\mathcal{U}$(0, 0.02)	$\mathcal{U}$(0, 0.1)
Extinction (e)	$\mathcal{U}$(0, 0.01)	$\mathcal{U}$(0, extinction)	$\mathcal{U}$(0, 0.1)
Interaction strength (A)	$\mathcal{U}$(-0.2, 0.2)	$\mathcal{U}$(-0.2, 0.2)	$\mathcal{U}$(-interaction, interaction)

Table 3. Parameter values of fixed parameters.

Parameter	Value
Number of individuals / lattice sites (I)	4000
Number of species (S)	100
Positive edge percentage (PEP)	20
Diagonal elements	-0.5
Connectance	0.02

The self-organized instability model can be reproduced by the stochastic generalized Lotka-Volterra model.

In the main text, we discussed that next to stochastic generalized Lotka-Volterra models there exists another algorithm to model interacting species stochastically, the individual-based model. We mention in particular the self-organized instability (SOI) model introduced by ***Solé et al.*** (***2002***). The question arises whether the SOI model is equivalent to the generalized Lotka-Volterra models, or if it can produce more or different characteristics. An elaborate study of the SOI has been performed in ***Faust et al.*** (***2018***). Most results are in agreement with our findings. However; in a supplemental figure of ***Faust et al.*** (***2018***) (S3), it is shown that the percentage of taxa with pink noise increases and the percentage of taxa with brown noise decreases when the stochasticity increases, where the stochasticity is defined as the ratio between the mean of extinctions and immigrations and the mean of the absolute interaction strengths (excluding diagonal values). This cannot be easily concluded from our previous results and we therefore studied this aspect in more detail.

We first evaluated the contributions of the different parameters. We therefore run simulations using the code provided by the authors of ***Faust et al.*** (***2018***), only changing one of the parameters at a time. What we notice is that the shift from brown to pink noise is mostly caused by the extinction rate and not the immigration rate or interaction strength (***Figure 22***, parameters are in ***Table 2*** and ***Table 3***).

In ***Figure 22***, we also see that with increasing extinction the mean abundance is decreasing (top panel). At the same time, the noise color is becoming lighter (shifting to pink). To understand the connection between the parameters, we made a figure with both the noise color and mean abundance on the axes for two time series (***Figure 23***A). We see that the noise color becomes more pink for smaller mean abundances. How do these results relate to the results for the generalized Lotka-Volterra equations?

There are multiple differences between the SOI and gLV models. First of all, the SOI model follows an individual-based approach. It is based on the Gillespie algorithm with the K-leap method. At every time step, the propensities for all events are calculated and K processes are chosen accordingly. The elapsed time τ is calculated by the gamma function. Since it is an individual-based model, the abundances will only take integer numbers. GLV models are continuous and the abundances of the time series are real positive numbers. We showed in the previous section that Rounding time series to integer numbers makes the noise color lighter.

A second difference between the SOI and our gLV model is that the SOI model is based on

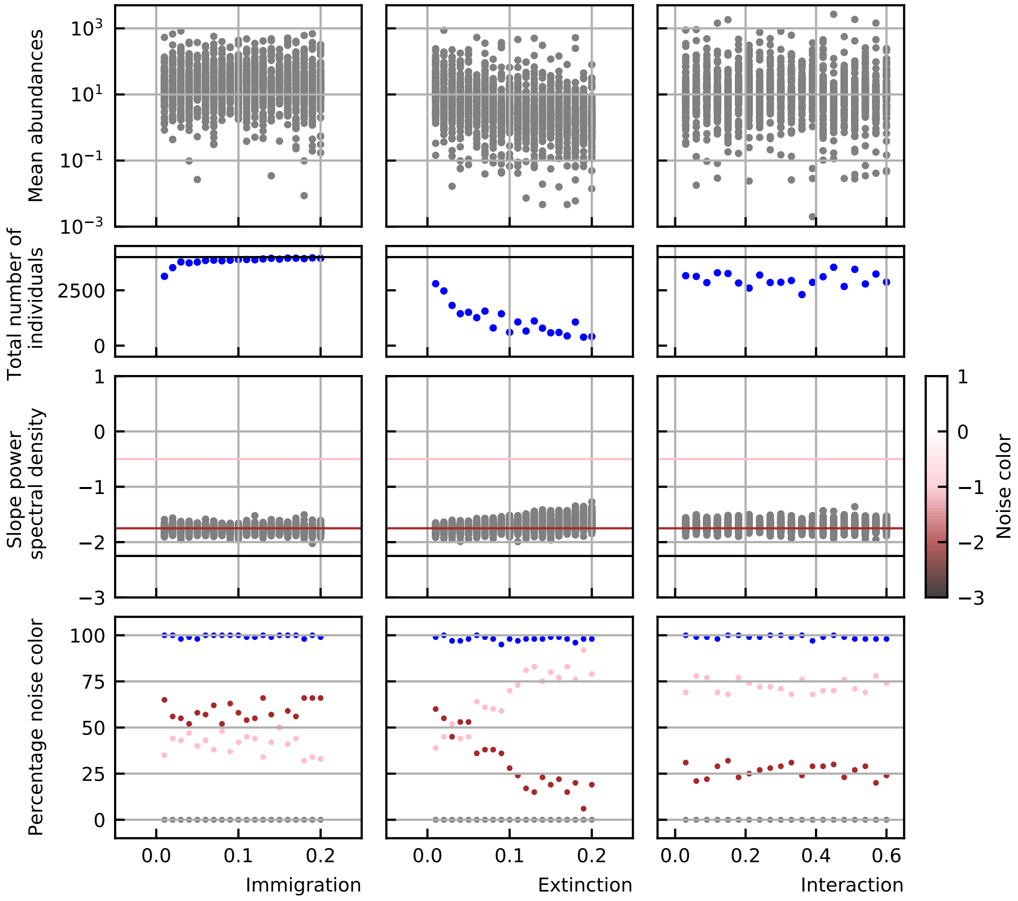

Figure 22. Time series simulations were performed for multiple values of the immigration, extinction and interaction rate. Shown are the mean abundance values of these simulations (top panels), the total number of individuals (second row), the distribution of the noise colors (third row) and the percentages of all noise colors as delimited by ***Faust et al.*** (***2018***) (bottom row). For the SOI model, an increase in the extinction rate causes a shift from brown to pink noise. At the same time, the mean abundances and total number of individuals decrease.

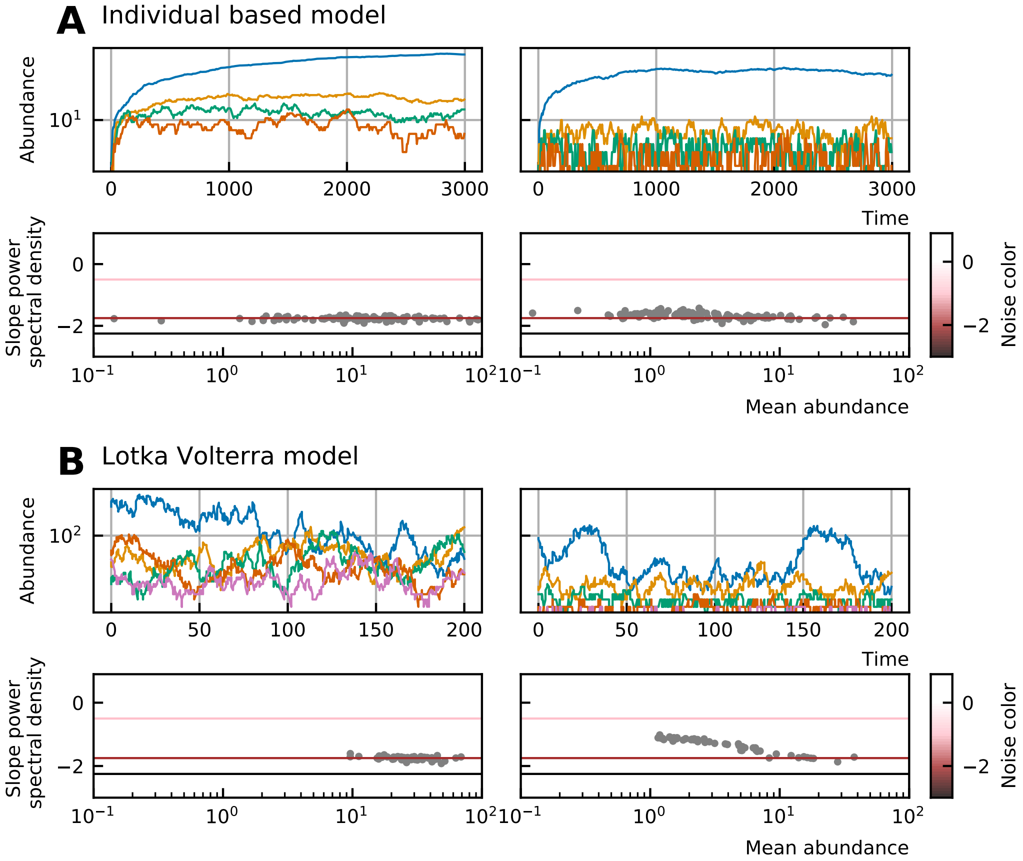

Figure 23. (A) Time series generated by the SOI model for different extinction rates, $e = 0.01$ for the left column and $e = 0.16$ for the right column (additional parameters can be found in the second column of ***Table 2*** and ***Table 3***). The lower figures show the noise color as a function of the mean abundance for the corresponding time series. For higher extinction (right side) the mean abundances are lower and the noise color becomes lighter with decreasing mean abundance. (B) Time series generated with the gLV model and rounded to integer precision after the simulation for different extinction rates, $e = 0.001$ on the left and $e = 0.8$ on the right. The noise color becomes lighter for low abundances.

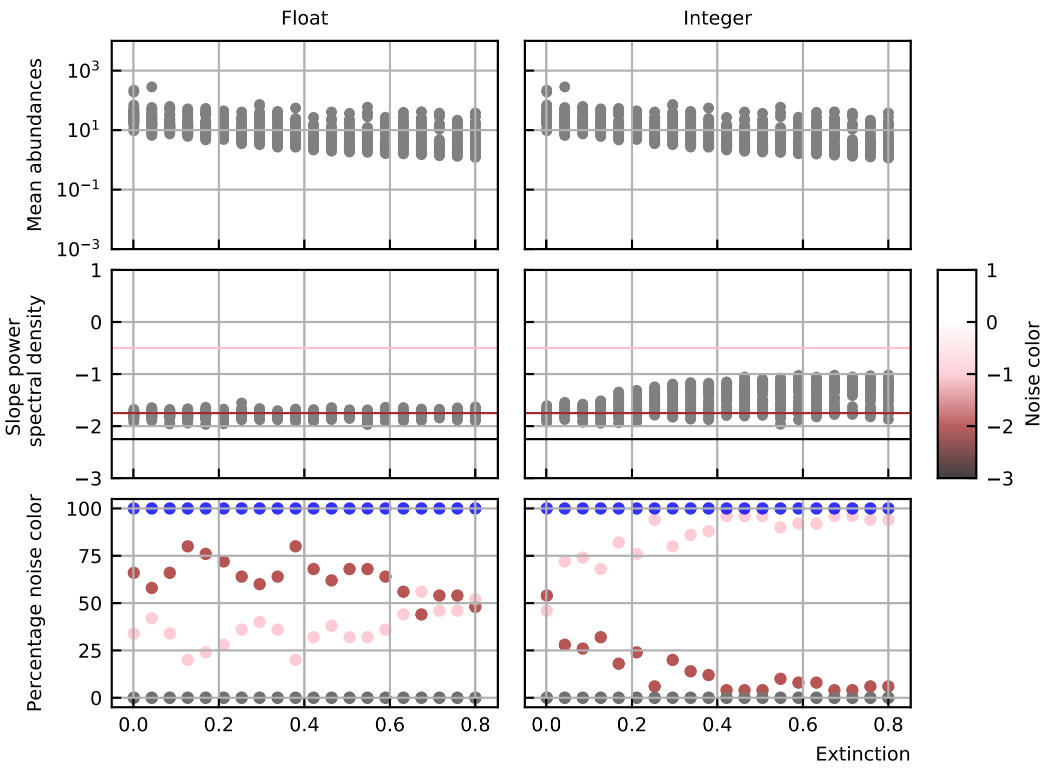

Figure 24. When imposing different constraints—addition of immigration, negative growth rates, small self-interaction, small precision—on the gLV model, the noise color shifts for increasing extinction as in the SOI model.

immigration and that no species can grow in the absence of other species. In the gLV model, we do not consider immigration and the growth rates of species are often positive. Furthermore, the interaction matrix is interpreted differently. Given the interaction matrix used for the SOI model (ω), the interaction matrix of the gLV model (ω') needs to obey the following rules for the time series to be comparable:

1. $\omega'_{ii} = 0$ all self-interactions are zero,
2. if $\omega_{ij} > \omega_{ji} > 0 : \omega'_{ij} = \omega_{ij} + \omega_{ji}$ and $\omega'_{ji} = 0$ mutualistic interactions are transformed into commensalistic interactions and,
3. if $\omega_{ij} < 0$ and $\omega_{ij} < \omega_{ji} : \omega'_{ij} = \omega_{ji} - \omega_{ij}$ and $\omega'_{ji} = 0$ competitive or parasitic interactions are transformed into amensalistic interactions.

The most important difference is that the self-interactions are zero. For gLV models, the self-interaction needs to be negative such that the abundance remains bounded and cannot go to infinity. In the SOI model, this constraint is imposed by the maximal number of individuals. The propensity for growth decreases linearly with the number of individuals. When the maximal number of individuals is attained all growth propensities are zero.

If we include all the aforementioned elements:

1. add immigration,
2. consider only negative growth rates (extinction),
3. small self-interaction,
4. round the results to integer values after performing the complete time series,

then we obtain similar results as the SOI model (***Figure 23***B and ***Figure 24***).

References

Arató M. A famous nonlinear stochastic equation (Lotka-Volterra model with diffusion). Mathematical and Computer Modelling. 2003 Oct; 38(7-9):709–726. https://linkinghub.elsevier.com/retrieve/pii/S0895717703900562, doi: 10.1016/S0895-7177(03)90056-2.

Caporaso JG, Lauber CL, Costello EK, Berg-Lyons D, Gonzalez A, Stombaugh J, Knights D, Gajer P, Ravel J, Fierer N, Gordon JI, Knight R. Moving pictures of the human microbiome. Genome Biology. 2011; 12(5):R50. http://genomebiology.biomedcentral.com/articles/10.1186/gb-2011-12-5-r50, doi: 10.1186/gb-2011-12-5-r50.

David LA, Materna AC, Friedman J, Campos-Baptista MI, Blackburn MC, Perrotta A, Erdman SE, Alm EJ. Host lifestyle affects human microbiota on daily timescales. Genome Biology. 2014; 15(7):R89. http://genomebiology.biomedcentral.com/articles/10.1186/gb-2014-15-7-r89, doi: 10.1186/gb-2014-15-7-r89.

Faust K, Bauchinger F, Laroche B, de Buyl S, Lahti L, Washburne AD, Gonze D, Widder S. Signatures of ecological processes in microbial community time series. Microbiome. 2018 Dec; 6(1):120. https://microbiomejournal.biomedcentral.com/articles/10.1186/s40168-018-0496-2, doi: 10.1186/s40168-018-0496-2.

Martin-Platero AM, Cleary B, Kauffman K, Preheim SP, McGillicuddy DJ, Alm EJ, Polz MF. High resolution time series reveals cohesive but short-lived communities in coastal plankton. Nature Communications. 2018 Dec; 9(1):266. http://www.nature.com/articles/s41467-017-02571-4, doi: 10.1038/s41467-017-02571-4.

MetaHIT Consortium, Qin J, Li R, Raes J, Arumugam M, Burgdorf KS, Manichanh C, Nielsen T, Pons N, Levenez F, Yamada T, Mende DR, Li J, Xu J, Li S, Li D, Cao J, Wang B, Liang H, Zheng H, et al. A human gut microbial gene catalogue established by metagenomic sequencing. Nature. 2010 Mar; 464(7285):59–65. http://www.nature.com/articles/nature08821, doi: 10.1038/nature08821.

MetaHIT Consortium (additional members), Arumugam M, Raes J, Pelletier E, Le Paslier D, Yamada T, Mende DR, Fernandes GR, Tap J, Bruls T, Batto JM, Bertalan M, Borruel N, Casellas F, Fernandez L, Gautier L, Hansen T, Hattori M, Hayashi T, Kleerebezem M, et al. Enterotypes of the human gut microbiome. Nature. 2011 May; 473(7346):174–180. http://www.nature.com/articles/nature09944, doi: 10.1038/nature09944.

Solé RV, Alonso D, McKane A. Self-organized instability in complex ecosystems. Philosophical Transactions of the Royal Society of London Series B, Biological Sciences. 2002 May; 357(1421):667–671. doi: 10.1098/rstb.2001.0992.

Turnbaugh PJ, Hamady M, Yatsunenko T, Cantarel BL, Duncan A, Ley RE, Sogin ML, Jones WJ, Roe BA, Affourtit JP, Egholm M, Henrissat B, Heath AC, Knight R, Gordon JI. A core gut microbiome in obese and lean twins. Nature. 2009 Jan; 457(7228):480–484. http://www.nature.com/articles/nature07540, doi: 10.1038/nature07540.

Walczak AM, Mugler A, Wiggins CH. Analytic Methods for Modeling Stochastic Regulatory Networks. In: Liu X, Betterton MD, editors. *Computational Modeling of Signaling Networks*, vol. 880 Totowa, NJ: Humana Press; 2012.p. 273–322. http://link.springer.com/10.1007/978-1-61779-833-7_13, doi: 10.1007/978-1-61779-833-7_13.

Wennekes PL, Rosindell J, Etienne RS. The Neutral—Niche Debate: A Philosophical Perspective. Acta Biotheoretica. 2012 Sep; 60(3):257–271. http://link.springer.com/10.1007/s10441-012-9144-6, doi: 10.1007/s10441-012-9144-6.
